# Dyslipidemia Among Diabetes Mellitus Patients: A Case-Control Study From a Tertiary Care Hospital in South India

**DOI:** 10.7759/cureus.35625

**Published:** 2023-02-28

**Authors:** VL Asha Latha, Sai Sirisha Devi Mondu, Mummareddi Dinesh Eshwar, Aryan Reddy Polala, Sadhana Nandanavanam, Saikrishna Dodda

**Affiliations:** 1 Biochemistry, Government Medical College, Kadapa, Kadapa, IND; 2 Internal Medicine, Mahavir Institute of Medical Sciences, Vikarabad, IND; 3 Internal Medicine, Navodaya Medical College Hospital & Research Center, Raichur, IND

**Keywords:** very low-density lipoprotein, low-density lipoprotein, high-density lipoprotein, triglycerides, total cholesterol, dyslipidemia, type 2 diabetes mellitus

## Abstract

Background

Diabetes mellitus (DM) is a chronic endocrine disease characterized by impaired glucose metabolism. Type 2 DM (T2DM) is an age-related disease that usually affects middle and older-aged people who suffer from increased blood glucose activities. Several complications are associated with uncontrolled diabetes that include abnormal lipid levels/dyslipidemia. This may predispose T2DM patients to life-threatening cardiovascular diseases. Therefore, it is essential to evaluate the activities of lipids among T2DM patients.

Methodology

A case-control study involving 300 participants was conducted in the outpatient department of medicine attached to Mahavir Institute of Medical Sciences, Vikarabad, Telangana, India. The study included 150 T2DM patients and the same number of age-matched controls. In this study, 5 mL of fasting blood sugar (FBS) was collected from each participant for the estimation of lipids (total cholesterol (TC), triacylglyceride (TAG), low-density lipoprotein-cholesterol (LDL-C), high-density lipoprotein-cholesterol (HDL-C), and very low-density lipoprotein-cholesterol (VLDL-C)) and glucose.

Results

The FBS levels among T2DM patients (211.6 ± 60.97 mg/dL) and non-diabetic individuals (87.34 ± 13.06 mg/dL) were significantly (p < 0001) different. Analysis of lipid chemistry that included TC (174.8 ± 38.28 mg/dL vs. 157.22 ± 30.34 mg/dL), TAG (173.14 ± 83.48 mg/dL vs. 133.94 ± 39.69 mg/dL), HDL-C (37.28 ± 7.84 mg/dL vs. 43.4 ± 10.82 mg/dL), LDL-C (113.44 ± 28.79 mg/dL vs. 96.72 ± 21.53 mg/dL), and VLDL-C (34.58 ± 19.02 mg/dL vs. 26.7 ± 8.61 mg/dL) revealed significant variations among T2DM and non-diabetic individuals. There was a 14.10% decrease in the activities of HDL-C among T2DM patients along with an increase in the activities of TC (11.18%), TAG (29.27%), LDL-C (17.29%), and VLDL-C (30%).

Conclusions

T2DM patients have demonstrated abnormal lipid activities/dyslipidemia compared to non-diabetic patients. Patients with dyslipidemia may be predisposed to cardiovascular diseases. Therefore, regular monitoring of such patients for dyslipidemia is extremely vital to minimize the long-term complications associated with T2DM.

## Introduction

Diabetes mellitus (DM) is a metabolic illness that presents as elevated blood glucose levels/hyperglycemia. There are two forms of DM that include type 1 DM and type 2 DM (T2DM), wherein the latter is an age-related disease. T2DM is the most common form that affects >90% of adults, and an estimated one in 11 people suffer from T2DM. Moreover, Asian countries including India and China have emerged as diabetes epicenters. This may possibly be attributed to urbanization, which has brought with it a sedentary lifestyle and abnormal food habits. Furthermore, T2DM patients are predisposed to morbidity and mortality associated with cardiovascular complications [1,2].

T2DM is a complex disease that may be attributed to insulin resistance, impaired insulin secretion, increased glucose production, and genetic causes. Other aspects such as diet, sedentary lifestyle, smoking, and alcohol consumption have been noted to contribute to T2DM [1]. T2DM can cause dyslipidemia which comprises elevated triglycerides/triacylglycerides (TAG) and low-density lipoprotein-cholesterol (LDL-C) and lowered high-density lipoprotein-cholesterol (HDL-C). Lipid abnormalities are prevalent in T2DM patients because insulin resistance or deficiency affects key enzymes and pathways in lipid metabolism [3].

Several previous studies have pursued linking blood glucose levels to serum lipid activities [4-6]. The etiology of dyslipidemia appears to be centered on the increased hepatic secretion of large TAG-rich very low-density lipoprotein-cholesterol (VLDL-C) and its impaired clearance [7]. Because persistent hyperglycemia promotes glycosylation of all proteins, particularly collagen and matrix proteins of the artery wall, endothelial cell dysfunction eventually leads to atherosclerotic cardiovascular disease (ASCVD), the most prevalent consequence of diabetes [8,9].

Understanding the relationship between serum lipid patterns and different phases of glucose intolerance is crucial for clinical and public health purposes, and such information can serve as the foundation to predict future diabetes and initiate preventive measures [10].

Moreover, the lipid parameters may be influenced by the nutritional habits prevalent in the respective geographical regions [10-12]. Given the scarcity of data on the activities of lipids in our area, we attempted to evaluate the lipid profile of T2DM patients and compare those with non-diabetic individuals.

## Materials and methods

This case-control study included 300 participants and was conducted in the outpatient department (OPD) of medicine attached to Mahavir Institute of Medical Sciences (MIMS), Vikarabad, Telangana, India. The study included 150 T2DM patients and the same number of age-matched controls. The study was approved by the ethical review committee of MIMS, Vikarabad, Telangana (MIMS/IEC/2021/68/March/2021) and was conducted between June and August 2021.

Inclusion and exclusion criteria

All patients who were diagnosed with T2DM and had volunteered to participate were included in the study. Patients with type 1 DM and those who were on insulin therapy, lipid-lowering drugs, and drugs that cause dyslipidemia were excluded from the study. Patients who were suffering from hypothyroidism, obesity, and chronic renal failure were also excluded.

Diagnostic criteria

Diabetes was diagnosed based on the current World Health Organization (WHO) diagnostic criteria for diabetes, which suggests fasting plasma glucose ≥126.13 mg/dL or two-hour plasma glucose ≥200 mg/dL while normal fasting blood glucose (FBG) was <100 mg/dL [13].

Dyslipidemia was defined using the National Cholesterol Education Program-Adult Treatment Panel III (NCEP-ATP III) criteria that include TC >200 mg/dL, LDL-C >130 mg/dL, HDL-C <40 mg/dL for males, <50 mg/dL for females, and TAG ≥150 mg/dL [14].

In this study, 5 mL of blood (3 mL drawn in a plane tube and 2 mL drawn in a sodium fluoride tube) was collected from each participant after overnight fasting for 12-14 hours for the estimation of lipids and glucose. All tubes for lipids were allowed to clot and then centrifuged at room temperature at 2,000 rotations per minute for 10 minutes. The serum was collected and stored for further analysis.

Blood glucose was estimated using the glucose oxidase-peroxidase method wherein 70-110 mg/dL was considered normal FBG. Cholesterol was estimated using the cholesterol oxidase peroxidase method. TAG was measured using the glycerol phosphate oxidase method. HDL-C was evaluated using the phosphotungstate precipitation method. LDL-C [TC-(HDL+TAG/5)] and VLDL-C (TAG/5) were analyzed using formulae.

Statistical analysis

The data collected were entered into a Microsoft Office 2019 Excel sheet (Microsoft® Corp., Redmond, WA, USA). The data were processed using the statistical software SPSS version 11.0 (IBM Corp., Armonk, NY, USA). The data were systematically represented in tables, and the results were interpreted as mean, percentage, and standard deviation. The Student’s t-test was applied to determine the significance of the results wherein a p-value <0.05 was considered statistically significant.

## Results

The mean age of the diabetic population was 49.7 ± 11.38 years (range = 32-69 years) and of the non-diabetic population was 45.96 ± 11.02 years (range = 25-66 years). The mean values of FBG in the non-diabetic population (87.34 ± 13.06 mg/dL) and T2DM patients (211.6 ± 60.97 mg/dL) were found to be statistically significant (p < 0.0001). Moreover, there was a significant (p < 0.0001) variation in the TC among T2DM patients (174.8 ± 38.28 mg/dl) and non-diabetic controls (157.22 ± 30.34 mg/dL). The details of the FBG and lipid parameters among T2DM patients and non-diabetic individuals are shown in Table 1.

**Table 1:** Comparison of FBG and lipid parameters among diabetic and non-diabetic subjects. *: significant p-values. T2DM: type 2 diabetes mellitus; FBG: fasting blood glucose; TC: total cholesterol; TAG: triacylglyceride; HDL-C: high-density lipoprotein-cholesterol; LDL-C: low-density lipoprotein-cholesterol; VLDL: very low-density lipoprotein-cholesterol

Parameter	T2DM patients (n = 150), mean ± SD	Non-diabetic subjects (n = 150), mean ± SD	P-value
FBG (mg/dL)	211.6 ± 60.97	87.34 ± 13.06	<0.0001*
TC (mg/dL)	174.8 ± 38.28	157.22 ± 30.34	<0.0001*
TAG (mg/dL)	173.14 ± 83.48	133.94 ± 39.69	<0.0001*
HDL-C (mg/dL)	37.28 ± 7.84	43.4 ± 10.82	<0.0001*
LDL-C (mg/dL)	113.44 ± 28.79	96.72 ± 21.53	<0.0001*
VLDL-C (mg/dL)	34.58 ± 19.02	26.7 ± 8.61	<0.0001*

Among T2DM patients, 74% had normal TC activities (<200 mg/dL), 18% showed borderline values (200-239 mg/dL), and 8% had high values of TC (≥240 mg/dL). Analysis of TAG activities among T2DM patients confirmed that 58% had normal activities (<100 mg/dL), 26% had borderline values (100-159 mg/dL), and 16% had high values (≥160 mg/dL). With reference to HDL-C activities, 42% had a normal value (<40 mg/dL), 56% had a borderline value (40-59 mg/dL), and 2% showed a high value (≥60 mg/dL). Serum HDL-C was on the lower side among T2DM patients compared to non-diabetic subjects. The detailed depiction of lipid parameters among T2DM patients and non-diabetic participants is shown in Table 2.

**Table 2:** Lipid profile of the study participants. T2DM: type 2 diabetes mellitus; TC: total cholesterol; TAG: triaglycerides; HDL-C: high-density lipoprotein-cholesterol; LDL-C: low-density lipoprotein-cholesterol; VLDL: very low-density lipoprotein-cholesterol

Parameters	Non-diabetic subjects (n = 150)	T2DM patients (n = 150)
TC (mg/dL)		
Normal (<200)	141 (94%)	111 (74%)
Borderline (200–239)	9 (6%)	27 (18%)
High (≥240)	0	12 (8%)
TAG (mg/dL)		
Normal (<100)	102 (68%)	87 (58%)
Borderline (100–159)	36 (24%)	39 (26%)
High (≥160)	12 (8%)	24 (16%)
HDL-C (mg/dL)		
Normal (<40)	36 (24%)	63 (42%)
Borderline (40–59)	105 (70%)	84 (56%)
High (≥60)	9 (6%)	3 (2%)
LDL-C (mg/dL)		
Normal (<40)	96 (64%)	96 (64%)
Borderline (100–159)	54 (36%)	51 (34%)
High (≥60)	0 (0)	3 (2%)
VLDL-C (mg/dL)		
Optimal (2–30)	111 (74%)	84 (56%)
High (≥31)	39 (26%)	66 (44%)

A significant increase in TAG and VLDL-C activities along with a moderate increase in the levels of TC and LDL-C was observed in T2DM patients compared to non-diabetic individuals. In a comparison with non-diabetic individuals, T2DM patients revealed a maximum increase (30% and 29.27%) in the activities of VLDL-C and TAG, respectively, followed by a high rise of LDL-C (17.29%) and TC (11.18%). In contrast, only HDL-C activities showed a decreasing (14.10%) trend among T2DM patients. Comparative variations in the lipid parameters among T2DM patients and non-diabetic subjects are shown in Table 3.

**Table 3:** Variation of lipid parameters among T2DM patients. T2DM: type 2 diabetes mellitus; TC: total cholesterol; TAG: triacylglyceride; HDL-C: high-density lipoprotein-cholesterol; LDL-C: low-density lipoprotein-cholesterol; VLDL: very low-density lipoprotein-cholesterol

Parameters	Variation in T2DM patients
TC (mg/dL)	Increase by 11.18%
TAG (mg/dL)	Increase by 29.27%
HDL-C (mg/dL)	Decrease by 14.10%
LDL-C (mg/dL)	Increase by 17.29%
VLDL-C (mg/dL)	Increase by 30%

## Discussion

According to a 2019 estimate, the prevalence of DM in India is approximately 77 million, with T2DM constituting 90% of all diabetic populations [15]. The prevalence of DM among the Indian population per the National Family Health Survey-4 and Longitudinal Aging Survey in India was noted to be 2.1% (95% confidence interval (CI) = 2.0-2.3%) and 1.7% (95% CI = 1.6-1.8%) among males (15-50 years) and females (15-49 years), respectively [16].

According to the results of a recent study, the death and disability-adjusted life years among T2DM patients showed a significant increase over the past two decades (1999-2019). Moreover, this trend was much more evident in the Asian (central and southern) and southern Sub-Saharan African regions [17]. T2DM patients suffer from complications arising from cardiovascular disease (CVD) and this is more evident among those who have other risk factors such as dyslipidemia, obesity, and hypertension among others [18].

The mechanisms underlying dyslipidemia among T2DM patients may be multifaceted. Despite many factors affecting lipid levels in diabetes, carbohydrate metabolism directly influences lipid metabolism. Furthermore, insulin deficiency causes higher metabolism of free fatty acids and causes lipid metabolism. Lipid metabolism plays an important role in increased vascular risk. Insulin resistance among T2DM patients may cause fat cells to break down from their stored TAG forms and result in a greater release of free fatty acids into the circulation. Increased fatty acids in the plasma lead to increase fatty acid uptake by the liver. The liver takes those fatty acids and synthesizes them back into TAGs. The presence of increased TAGs stimulates the secretion and assembly of the apolipoprotein B and VLDL-C [19].

Coronary artery disease (CAD)/CVD is generally attributed to atherosclerosis of large and medium-sized arteries, and diabetic dyslipidemia has been reported as one of the most important contributing factors [18,20,21].

Previous studies have noted that an increase in the activities of LDL-C and dyslipidemia may predispose T2DM patients to coronary heart disease and can predict future cardiovascular complications [20,21]. The findings of this study demonstrated an increase in TC by 11.8% in T2DM patients compared to the non-diabetic population. Similar findings were noted in previous studies from Turkey and India [19,22]. An increase in TC may be due to cholesterol absorption deficiency and higher cholesterol synthesis, especially among obese persons with diabetes, which suggests that diabetes modulates lipid metabolism [23,24].

A significant increase (p = 0003) in the mean TAG in T2DM (29.27% increase) compared to the non-diabetic population was observed in this study. A similar trend was noted in a study from China where an increase of 19.9% was observed [25].

A remarkable decrease (72% decrease) in the activities of HDL-C was noted in a previous study as against our results which demonstrated a 14.10% decrease in HDL-C activities [26]. Lower HDL-C activities in T2DM may be due to reduced lipoprotein lipase (LPL) activity. The low LPL activity may affect the increase in HDL-C. The normal insulin-mediated stimulation of LPL activities is interrupted by insulin resistance [25].

An elevation in the activities of LDL-C (17.29% increase) and VLDL-C (30% increase) was observed in the present study which was in agreement with other previous studies [22,27,28].

T2DM has proven to be a predisposing factor for the development of ASCVD. The elevated activities of LDL-C fraction can result in ASCVD, and, therefore, it is crucial to track the lipid levels among T2DM patients and initiate effective management strategies to slow the progression and minimize the complications [29,30].

Study limitations

This study was conducted in a single center in South India. Moreover, the participants in the study were not evaluated/categorized based on diet, genetics, and other physiological and demographic factors which could have influenced the results of the study. Furthermore, this is a point-prevalence study, and, therefore, the results obtained may not truly reflect individual biochemical characteristics.

## Conclusions

The present study findings concluded that there is an increase in blood glucose and lipid parameters with a significant rise observed in TC, TAG, and LDL-C, and a substantial decrease in HDL-C activities in T2DM patients when compared to the non-diabetic population. Among the lipid parameters, VLDL-C and TAG showed the maximum rise, followed by LDL-C and TC activities. Moreover, the percentage increase in VLDL-C correlated with the increase in TAG and TC. T2DM is becoming an epidemic in many countries, and the average age of onset of T2DM is decreasing over time. The global burden of CVD/CAD in the world is huge, and the majority of those exist in developing countries. Despite the availability of pharmacological therapy such as statins, they are often associated with side effects and the therapy is lifelong and expensive. There is a possibility to prevent the onset of T2DM by early detection of glucose and cholesterol levels in the blood. Hence, there is a need for frequent health checkups to monitor the lipid parameters in the diabetic population and those who could be predisposed to T2DM. Regular monitoring of blood lipids may prevent the progression of CVD/CAD. Data on lipid profiles can assist the physician in recommending lifestyle and other changes, such as diet regulations and exercise, to reverse the changes, and, thus, decrease the chances of cardiovascular events such as myocardial infarctions and stroke.
